# Editorial: Direct or indirect endocrine and metabolic consequences of malignancies

**DOI:** 10.3389/fendo.2024.1507071

**Published:** 2024-11-26

**Authors:** Chen Wu, Oluf Dimitri Røe, Jia Wei

**Affiliations:** ^1^ Department of Oncology, First People’s Hospital of Changzhou City, Changzhou, China; ^2^ Department of Oncology, The 3^rd^ Affiliated Hospital of Soochow University, Changzhou, China; ^3^ Department of Clinical and Molecular Medicine, Norwegian University of Science and Technology (NTNU), Trondheim, Norway; ^4^ The Comprehensive Cancer Centre of Nanjing Drum Tower Hospital, Nanjing, China

**Keywords:** malignance, immune checkpoint inhabitor, endocrine and metabolic consequences, direct, indirect

**Figure 1:**
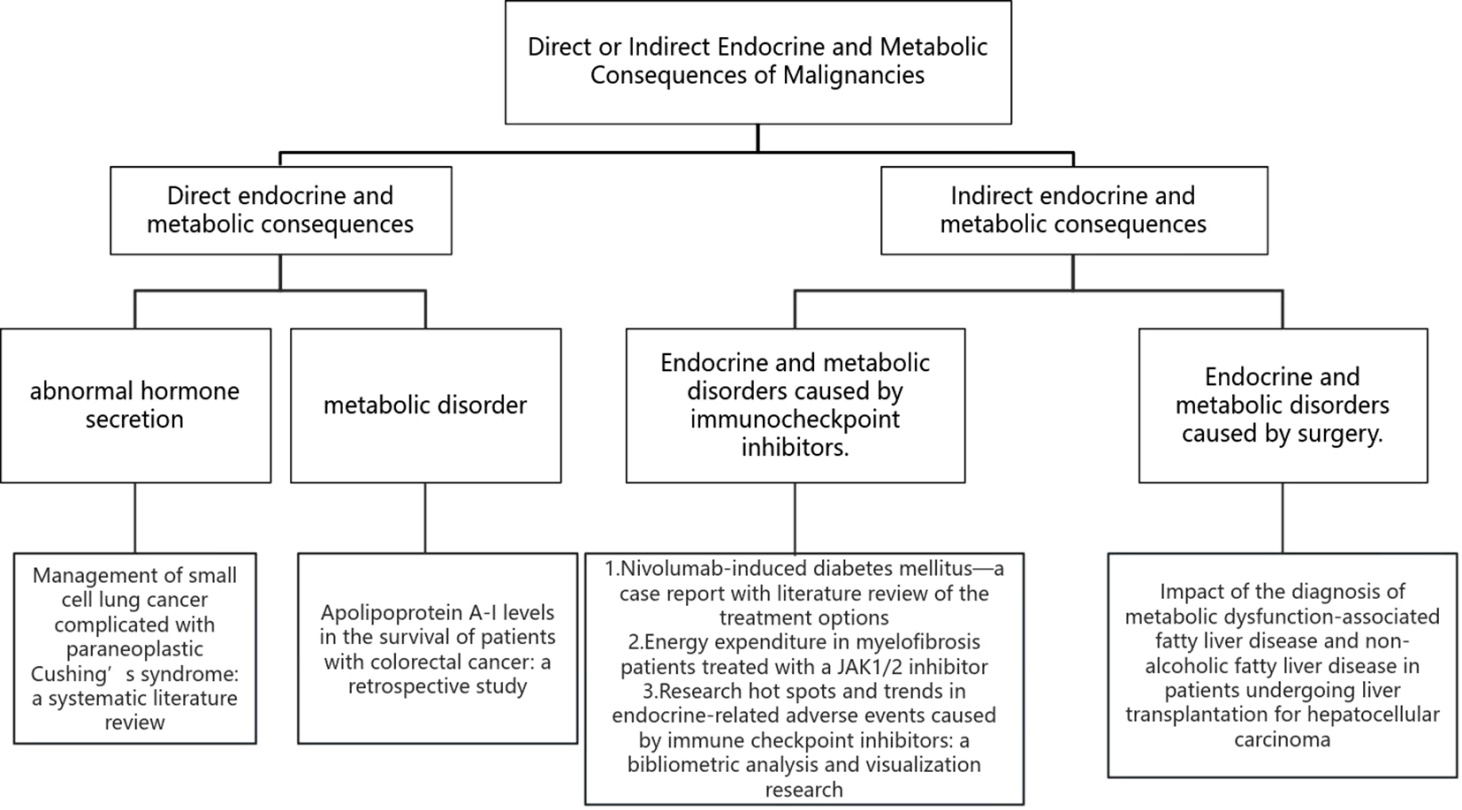
Direct or indirect endocrine and metabolic consequences of malignancies.

Malignancies of endocrine organs, such as the pituitary, thyroid, parathyroid, and adrenal glands, significantly affect the metabolism of patients, leading to poor prognosis and high mortality; In addition, malignancies of other organs (such as the pancreas and lung) also cause endocrine or metabolic syndromes, such as Cushing’s syndrome, hypoglycemia, hypercalcemia, male mammogenesis, which are called para-neoplastic syndrome. The above mentioned situations are named direct endocrine consequences of malignancies (**Figure 1**). Moreover, the commonly used chemo-, targeted- and immune therapeutic agents (especially immunocheckpoint inhibitors) also have non-negligible impacts on endocrine homeostasis, sometimes life-threatening, through various potential mechanisms, which is named indirect endocrine consequences of malignancies.

Among the 9 publications on this Research Topic, one article is a case report, one is a mini-review, one is a systematic review, and six are original research articles. Among them, two describe the direct impact of malignant tumors on endocrine and metabolism, and four describe the impact of immunotherapy for malignant tumors on endocrine and metabolism. Regarding the direct impact of malignant tumors on endocrine function, Li et al. summarized the pathogenesis and differential diagnosis of paraneoplastic Cushing’s syndrome(PCS) caused by small cell lung cancer (SCLC) and proposed a differential method for identifying PCS through immunohistochemical (IHC) staining. SCLC can directly secrete adrenocorticotropic hormone (ACTH) and corticotropin-releasing hormone (CRH). Abnormal secretion of ACTH and CRH can lead to PCS, which is reported to account for 1.6%-6% of all SCLC cases but has a worse prognosis among all SCLC patients. Regarding the direct impact of malignant tumors on metabolism, Xie et al. proposed that serum lipoprotein A-I levels are associated with the prognosis of patients with colorectal cancer(CRC).The article discusses that serum apolipoprotein A-I levels decrease in CRC patients, and patients with lower serum apolipoprotein A-I levels have lower progression-free survival and overall survival than those with higher levels. In addition, treatment of malignant tumors also affects patient metabolism, Zhu et al. mentioned that the incidence of metabolic dysfunction-related fatty liver disease(MAFLD) and non-alcoholic fatty liver disease (NAFLD) increases in patients with hepatocellular carcinoma(HCC) after liver transplantation surgery. The diagnosis of MAFLD is more strongly associated with metabolic abnormalities than the diagnosis of NAFLD. Regarding the impact of immunotherapy for malignant tumors on metabolism, Tremblay et al. elucidated that weight gain in patients with myelofibrosis treated with the JAK1/2 inhibitor ruxolitinib may be related to changes in systemic energy expenditure. The JAK1/2 inhibitor ruxolitinib has been found to affect patient metabolism during the treatment of myelofibrosis, resulting in weight gain. This weight gain is not only related to changes in appetite due to impaired hypothalamic JAK/STAT signaling but also to changes in overall energy expenditure. As for the impact of immunotherapy for malignant tumors on endocrine function, Daetwyler et al. reported a case of diabetes induced by nivolumab monotherapy and suggested the use of insulin therapy as early as possible in the treatment of diabetes induced by immune checkpoint inhibitors, because treatment with infliximab failed to improve β-cell function, but insulin treatment was effective. Additionally, immune checkpoint inhibitors can produce various endocrine toxicities during treatment, Zhao et al. summarized the research hotspots and trends of endocrine-related adverse events caused by immune checkpoint inhibitors in recent years, identify the current research hotspots include the management of endocrine-related adverse events, hypophysitis, thyroid dysfunction, type I diabetes mellitus, and the impact of endocrine adverse events on survival of patients in this field. In addition, Dong et al. described the relationship between thyroid dysfunction and the risk of cutaneous malignant melanoma(CMM), and indicated that hypothyroidism might be a protective factor for CMM. Lai et al. described a causal relationship between hypothyroidism and rheumatoid arthritis(RA), while no causal relationship was found between hyperthyroidism and RA. Wang et al. described the relationship between hypothyroidism and endometrial cancer(EC) and indicated through MR analysis that there is a lack of causal relationship between hypothyroidism and EC.

From these nine articles, we can gain insight into the impact of malignant tumors on the body’s metabolism and endocrine function, which can predict patient prognosis. For instance, the article mentions that serum apolipoprotein A-I in CRC patients can be used as an effective biomarker for patient prognosis. In SCLC, PCS can be used as one of the prognostic indicators for patients. Similarly, MAFLD in HCC liver transplant patients can be used as an independent predictor of a high risk of HCC recurrence. For early identification of endocrine disorders in malignant tumors, IHC staining can be used as an effective diagnostic tool for distinguishing between PCS and Cushing’s disease. It is recommended that in the future, the application of IHC staining for unique hormones (ACTH or CRH) should be strengthened in clinical practice for early differential diagnosis of PCS. In addition, the treatment of diabetes caused by immune checkpoint inhibitors may differ from other immune-related adverse events. While other immune adverse events are typically treated with immunosuppressants, this approach is often ineffective in diabetes, and early use of insulin is the key to treatment. Furthermore, it should be noted that most endocrine adverse reactions caused by immunotherapy are due to the involvement of a single gland. However, with the gradual increase in the clinical use of immune checkpoint inhibitors, reports of involvement of two or more glands are becoming more common. According to studies, the combination of thyroid and pancreatic injury is the most common multi-gland injury. Patients receiving immunocheckpoint inhibitor therapy must continue to monitor these endocrine-related events. It is also crucial to actively seek effective biomarkers in clinical practice to predict the risk of endocrine adverse reactions, which will help early detection and management of patients’ immune adverse reactions.

